# Retraction and republication—Worldwide burden of cancer attributable to diabetes and high body-mass index: a comparative risk assessment

**DOI:** 10.1016/S2213-8587(18)30146-3

**Published:** 2018-06

**Authors:** The Editors of The Lancet Diabetes & Endocrinology

**Affiliations:** a The Lancet Diabetes & Endocrinology, London EC2Y 5AS, UK

On Nov 28, 2017, *The Lancet Diabetes & Endocrinology* published online a comparative risk assessment that estimated the worldwide burden of cancer attributable to diabetes and high body-mass index, and the Article was published in print in the February, 2018, issue of the journal.^1^ On Jan 22, 2018, the authors notified us that the relative risk for mortality for colorectal cancer from one of the study's sources^2^ had been inadvertently used instead of the relative risk for incidence. The Editors of *The Lancet Diabetes & Endocrinology* discussed the corrections that were needed in the paper and appendix as a result of this mistake. Our decision, in accordance with the Committee on Publication Ethics' guidelines, that because of the extent of the changes necessary, the previous version of the Article should be retracted and a corrected version republished after re-analysis and re-review.

Today we retract the previous version and republish online the corrected version of the Article and appendix,^3^ in which the findings have changed slightly—eg, some absolute numbers of cancers attributable to diabetes and high body-mass index have been altered. The overall message remains the same. The previous version of the Article has been added to the appendix in the new version and is marked retracted.
