# Supplementary material for: Worldwide burden of cancer attributable to diabetes and high body-mass index: a comparative risk assessment
Source: Lancet Diabetes Endocrinol. 2018 Feb;6(2):95–104. doi: 10.1016/S2213-8587(17)30366-2 (PMC5805864; doi:10.1016/S2213-8587(17)30366-2)
Supplement: Supplementary appendix [file mmc1.pdf]

THE LANCET
Diabetes & Endocrinology

Supplementary appendix

This appendix formed part of the original submission and has been peer reviewed. We post it as supplied by the authors.

Supplement to: Pearson-Stuttard J, Zhou B, Kontis V, Bentham J, Gunter M J, Ezzati M, et al. Worldwide burden of cancer attributable to diabetes and high body-mass index: a comparative risk assessment. *Lancet Diabetes Endocrinol* 2017; published online Nov 28. http://dx.doi.org/10.1016/S2213-8587(17)30366-2.

Appendix

Relative risks

For BMI, only cancers suggested by the World Cancer Research Fund (WCRF) in their continuous update project[1-10] as having strong evidence to be convincingly associated with excess BMI were included in the analysis. These were colorectal, gallbladder, pancreas, kidney, liver, oesophageal adenocarcinoma, corpus uteri, post-menopausal breast, ovarian, stomach cardia, multiple myeloma and thyroid cancer.

WCRF has not assessed the evidence for diabetes as a causal risk factor of site-specific cancers. We therefore reviewed the published literature that had reviewed causal associations between diabetes and site-specific cancers. We selected a meta-analysis identifying most convincing evidence, rigorous adjustment and accounting for bias and heterogeneity.[11] This meta-analysis found sufficient evidence to conclude causal associations of diabetes with liver, pancreas, gallbladder, colorectal, breast and corpus uteri cancers.

Appendix Table 1. **List of cancers associated with overweight and obesity and diabetes included in this study and relative risks (RRs).**

**Cancer**	**RR due to High BMI**	**RR due to Diabetes**
Oesophageal (adenocarcinoma) (C15)	1.48 (1.35-1.62)[10]	
Colorectal (C18-21)	1.10 (1.10-1.16)[2]	1.20 (1.03-1.40)[11]
Gallbladder (C23)	1.25 (1.15-1.37)[3]	1.52 (1.26-1.84)[11]
Pancreatic (C25)	1.10 (1.07-1.14)[4]	1.95 (1.66-2.28)[11]
Postmenopausal Breast (C50)	1.13 (1.08-1.18)[1]	
Breast (C50)		1.20 (1.12-1.28)[11]
Corpus uteri (endometrial) (C54)	1.50 (1.42-1.59)[5]	1.97 (1.71-2.27)[11]
Ovarian (C56)	1.06 (1.02-1.11)[6]	
Kidney (C64)	1.30 (1.25-1.35)[7]	

Stomach Cardia (C16)	1.23 (1.07-1.40)[9]	
Liver (C22)	1.30 (1.16-1.46)[8]	2.31 (1.87-2.84)[11]
Multiple Myeloma (C88 & 90)	1.11 (1.05-1.18)[12]	
Thyroid (C73)	1.10 (1.00-1.10)[13]	

All RRs had the same mean and distribution across all age, gender and country groups, with the exception of endometrial, ovarian and breast cancers which were only for women. High BMI is associated with post-menopausal breast cancer hence this RR was applied to women 50 years and over only, whereas diabetes is associated with breast cancer across all ages.

Cancer data

As incidence of oesophageal and stomach cancers were not reported by histological or anatomical subtype in GLOBOCAN, but WCRF deemed a convincing association with oesophageal adenocarcinoma and stomach cardia only, the number of these cancers globally in 2012 was estimated based on region and sex-specific proportions reported in Cancer Incidence in Five Continents Volume X (C15 X)[14]. Here we calculated the proportions of the cancer sub-type of all site-specific cancers, i.e. proportion of all oesophageal cancer incidence that were adenocarcinoma and proportion of all stomach cancer that were stomach cardia. We then aggregated the proportions by region, sex and age. We assessed these proportions and identified heterogeneity by region and sex. We therefore applied these proportions to GLOBOCAN incidence data stratified by region and sex, for the corresponding cancer site (oesophageal or stomach) and multiplied the incidence data by the proportion to obtain incidence in 2012 of each cancer sub-type in each stratum. Diabetes and BMI data for age bands between 18 and 39 years were combined by using population weights to correspond with the GLOBOCAN age band of 15-39 years.

Calculation of population attributable fraction (PAF)

The methods we use to estimate PAFs are outlined in the main text. The PAF attributable to diabetes and high BMI individually was calculated using the following formula[15]:

$$\mathbf{PAF} = \frac{\sum P_i RR_i \; - \; \sum P'_i RR_i}{\sum P_i RR_i}$$

where P_i is the actual prevalence of diabetes or BMI category, P'_i is the prevalence in an alternative scenario and RR_i is the relative risk of site-specific cancer associated with diabetes or high BMI. Our main analyses to estimate PAF used current risk factor prevalence relative to an optimal prevalence as the alternative scenario in the first instance to measure the total cancer burden of diabetes and high BMI. We used zero diabetes prevalence and 20-25 kg/m^2 as the optimal BMI range of (used as 22.5 kg/m^2 in the calculation) where the cancer risk is assumed to be lowest at the population level.

When modelling the conservative combined scenario, as described in the manuscript we selected the larger of $PAF_{Diabetes}$ and $PAF_{High\ BMI}$ in each age, gender and country stratum to generate a conservative PAF.

Uncertainty analysis

We propagated the uncertainties of diabetes and categorical BMI prevalence and RRs to the final estimates using a simulation approach. We could not include the uncertainty in cancer incidence data because GLOBOCAN does not provide any measure of uncertainty. Specifically, we generated 1,000 draws for each RR from a log-normal distribution, with mean equal to the reported estimate and standard deviation calculated using the reported confidence interval. In the infrequent event that the conservative RR was below 1.0, we

limited this to have 1.0 as the minimum RR. We further used 1,000 draws from the posterior distributions of diabetes and BMI prevalence estimates from a Bayesian hierarchical model which pooled population-based studies and was fitted using a Markov chain Monte Carlo algorithm. We used the log-normal distribution for the RRs because the vast majority of RRs were estimated using models with exponential links such as Poisson and proportional hazard models. The coefficients of treatment or exposure in the models, which have been shown to have a normal distribution (through bootstrapping), are the logarithm of the RR and therefore the RR has a log-normal distribution. The PAF calculation was repeated for each of these draws resulting in 1,000 PAFs and attributable cases which characterised the uncertainty distribution of the outputs. We reported 95% uncertainty intervals for our estimates as the 2.5^{th}-97.5^{th} percentiles of the results. All statistical analyses were conducted using R version 3.2.5.

Appendix Results:

Appendix Table 2: **Population attributable fraction (PAF) of site-specific cancers attributable to diabetes and high BMI prevalence, individually and combined under the independent and conservative scenarios, in 2024 and 2035.**

	2024				2035			
	**High BMI PAF**	**Diabetes PAF**	**Independent PAF**	**Combined PAF**	**High BMI PAF**	**Diabetes PAF**	**Independent PAF**	**Conservative PAF**
**Men**								
Colorectal	5.9%	2.8%	8.5%	6.1%	7.3%	3.5%	10.5%	9.2%
Gallbladder	14.6%	7.9%	21.3%	15.2%	17.8%	9.5%	25.5%	17.6%
Liver	16.2%	15.6%	28.9%	19.0%	19.1%	18.6%	34.3%	25.5%
Pancreas	6.1%	13.3%	18.5%	13.5%	7.6%	16.0%	22.3%	17.5%
Kidney	16.8%		16.8%	16.8%	20.5%		20.5%	20.5%
Oesophagus (Adenocarcinoma)	26.2%		26.2%	26.2%	31.3%		31.3%	31.3%
Stomach (Cardia)	14.1%		14.1%	14.1%	17.1%		17.1%	17.1%
Multiple Myeloma	7.0%		7.0%	7.0%	8.7%		8.7%	8.7%
Thyroid	5.7%		5.7%	5.7%	7.2%		7.2%	7.2%
**Women**								
Breast	6.2%	2.1%	8.0%	6.5%	7.0%	2.7%	9.4%	9.8%
Endometrial	34.4%	12.3%	42.1%	34.4%	39.5%	14.6%	47.9%	40.2%
Colorectal	7.7%	2.8%	10.3%	7.8%	9.1%	3.3%	12.1%	9.5%
Gallbladder	19.1%	8.1%	25.5%	19.2%	22.1%	9.3%	29.2%	18.6%
Liver	21.5%	16.4%	33.9%	22.5%	24.9%	18.7%	38.5%	24.4%
Pancreas	8.0%	13.1%	20.0%	13.5%	9.5%	15.1%	22.9%	15.3%
Kidney	21.2%		21.2%	21.2%	24.6%		24.6%	24.6%
Ovarian	4.5%		4.5%	4.5%	5.4%		5.4%	5.4%
Oesophagus (Adenocarcinoma)	33.0%		33.0%	33.0%	37.3%		37.3%	37.3%

Stomach (Cardia)	17.4%		17.4%	17.4%	20.2%		20.2%	20.2%
Multiple Myeloma	9.5%		9.5%	9.5%	11.1%		11.1%	11.1%
Thyroid	7.0%		7.0%	7.0%	8.5%		8.5%	8.5%

PAFs in 2024 and 2035 due to diabetes and high BMI prevalence in 2014 and 202 5 respectively. 2014 estimates and 2025 prevalence projections based on past trends for diabetes[16] and high BMI[17,18].

Appendix Table 3: **Age Standardised prevalence of diabetes (%), mean body mass index (BMI) and BMI categorical prevalence (%) in 1980 and 2012 by region from recent published estimates by NCD RisC Collaboration[16,17].** A) Female B) Male.

A)

				BMI Categories						
**Female**	**Year**	**Diabetes**	**Mean BMI**	**<18.5**	**18.5-20**	**20-25**	**25-30**	**30-35**	**35-40**	**40+**
Central and eastern Europe	1980	6.6 (2.9-12.6)	25.5 (24.7-26.3)	3.0 (1.8-4.5)	6.9 (4.8-9.4)	42.3 (36.5-48.0)	29.0 (24.0-35.0)	14.0 (10.2-18.8)	3.7 (2.2-6.0)	1.1 (0.5-2.1)
Central Asia and north Africa-Middle East	1980	6.4 (2.911.4)	24.7 (23.7-25.6)	6.2 (3.8-9.4)	9.5 (6.2-14.3)	42.5 (35.4-49.7)	27.2 (21.5-33.9)	11.6 (7.9-16.1)	2.6 (1.4-4.3)	0.6 (0.2-1.2)
East and south east Asia	1980	4.9 (1.8-10.4)	21.1 (20.6-21.7)	16.2 (12.5-20.4)	19.4 (15.1-23.9)	51.8 (46.7-56.9)	11.5 (8.8-14.5)	1.1 (0.6-1.7)	0.0 (0.0-0.1)	0.0 (0.0-0.0)
High-income Asia Pacific	1980	5.2 (2.6-9.0)	22.0 (21.7-22.3)	9.8 (7.6-12.1)	16.5 (13.5-19.7)	57.2 (53.2-60.9)	14.9 (12.5-17.5)	1.5 (1.0-2.2)	0.1 (0.1-0.1)	0.0 (0.0-0.0)
High-income western countries	1980	4.5 (2.4-7.4)	24.7 (24.4-25.0)	3.6 (2.7-4.7)	7.8 (6.2-9.5)	49.1 (45.4-52.7)	26.2 (23.0-29.6)	9.8 (7.8-12.0)	2.6 (1.8-3.5)	0.9 (0.5-1.3)
Latin America and the Caribbean	1980	5.9 (2.8-10.7)	23.7 (22.8-24.5)	5.9 (3.8-8.7)	8.3 (5.7-11.7)	45.1 (39.4-51.0)	28.3 (23.5-33.5)	9.9 (7.0-13.3)	2.1 (1.1-3.3)	0.4 (0.2-0.8)
Oceania	1980	6.8 (3.1-12.9)	22.5 (21.0-24.1)	7.8 (3.0-15.8)	10.0 (4.7-17.7)	44.5 (33.8-55.5)	25.2 (17.5-34.4)	9.3 (5.0-15.4)	2.5 (1.1-5.4)	0.6 (0.2-1.4)
South Asia	1980	4.6 (1.8-9.4)	19.0 (18.4-19.6)	34.9 (28.3-41.9)	19.8 (14.4-25.6)	37.2 (30.4-43.9)	7.2 (4.7-10.0)	0.8 (0.4-1.4)	0.1 (0.0-0.1)	0.0 (0.0-0.0)
Sub-Saharan Africa	1980	3.3 (1.4-6.8)	20.7 (19.9-21.4)	16.7 (11.8-22.0)	19.3 (14.4-24.6)	47.4 (41.5-53.1)	12.9 (9.7-16.7)	2.8 (1.8-4.1)	0.6 (0.3-1.1)	0.2 (0.1-0.5)
Central and eastern Europe	2012	7.3 (4.5-10.9)	26.4 (26.0-26.9)	2.2 (1.6-3.0)	5.3 (4.1-6.7)	37.5 (33.8-41.3)	29.8 (26.3-33.1)	16.6 (13.5-19.8)	6.3 (4.6-8.1)	2.4 (1.5-3.6)
Central Asia and north Africa-Middle East	2012	13.7 (11.1-16.7)	28.1 (27.9-18.3)	2.9 (2.3-3.5)	3.9 (3.1-4.6)	29.1 (26.5-31.7)	31.4 (28.9-34.0)	20.6 (18.3-23.1)	8.2 (6.8-9.7)	3.9 (3.0-4.9)
East and south east Asia	2012	7.4 (5.2-10.2)	23.3 (23.1-23.6)	8.6 (7.1-10.4)	11.7 (9.6-14.0)	51.0 (47.5-54.3)	22.5 (19.8-25.3)	5.3 (4.1-6.6)	0.8 (0.5-1.1)	0.2 (0.1-0.3)
High-income Asia Pacific	2012	5.6 (4.0-7.5)	22.3 (22.1-22.5)	8.0 (6.3-10.1)	14.2 (11.8-16.9)	54.4 (50.9-57.8)	19.5 (16.8-22.1)	3.4 (2.6-4.4)	0.4 (0.3-0.6)	0.0 (0.0-0.0)
High-income western countries	2012	5.2 (4.0-6.8)	26.9 (26.7-27.1)	1.8 (1.4-2.3)	4.9 (4.1-5.8)	36.3 (34.0-38.7)	29.3 (27.1-31.5)	15.6 (3.7-17.6)	7.2 (5.9-8.6)	4.9 (3.7-6.3)
Latin America and the Caribbean	2012	9.3 (6.8-12.3)	27.1 (26.9-27.3)	2.5 (1.9-3.1)	4.4 (3.5-5.4)	33.3 (30.5-36.2)	32.7 (30.0-35.3)	17.5 (15.3-19.7)	7.0 (5.7-8.5)	2.7 (2.0-3.5)
Oceania	2012	14.9 (9.4-22.2)	26.5 (25.6-27.4)	2.8 (1.2-5.6)	4.2 (2.3-7.1)	32.9 (26.4-39.8)	32.3 (26.2-38.8)	17.4 (12.0-23.8)	7.2 (4.1-11.6)	3.2 (1.6-5.8)
South Asia	2012	8.7 (5.8-12.2)	22.0 (21.7-22.3)	24.0 (20.5-27.8)	14.4 (11.7-17.4)	40.5 (36.4-44.4)	16.2 (13.7-19.0)	3.9 (2.9-5.1)	0.8 (0.5-1.1)	0.2 (0.1-0.4)
Sub-Saharan Africa	2012	6.6 (4.9-8.6)	23.9 (23.7-24.1)	10.1 (8.5-11.9)	11.4 (9.9-13.1)	42.8 (40.3-45.3)	22.8 (20.8-24.9)	8.5 (7.3-9.7)	2.8 (2.3-3.4)	1.7 (1.3-2.1)

B)

Male		Diabetes	Mean BMI	<18.5	18.5-20	20-25	25-30	30-35	35-40	40+
Central and eastern Europe	1980	5.2 (2.2-10.1)	24.6 (23.9-25.2)	2.1 (1.2-3.2)	6.2 (4.4-8.7)	49.7 (43.9-55.2)	34.2 (28.8-39.9)	7.1 (4.9-9.9)	0.7 (0.3-1.4)	0.1 (0.0-0.2)
Central Asia and north Africa-Middle East	1980	5.5 (2.5-10.1)	23.4 (22.6-24.2)	6.6 (4.1-10.1)	9.6 (6.6-13.4)	50.3 (43.3-57.5)	28.0 (22.0-34.7)	5.0 (3.1-7.5)	0.4 (0.2-0.8)	0.0 (0.0-0.1)
East and south east Asia	1980	3.5 (1.2-7.9(	20.9 (20.4-21.4)	13.4 (10.0-17.4)	21.0 (16.5-25.8)	55.6 (50.2-61.0)	9.5 (6.8-12.7)	0.4 (0.2-0.6)	0.0 (0.0-0.0)	0.0 (0.0-0.0)
High-income Asia Pacific	1980	5.5 (2.7-9.3)	22.1 (21.8-22.4)	7.7 (5.9-9.7)	15.3 (12.6-18.3)	60.6 (57.0-64.1)	15.5 (12.9-18.3)	0.8 (0.5-1.1)	0.0 (0.0-0.0)	0.0 (0.0-0.0)
High-income western countries	1980	4.8 (2.6-8.0)	25.1 (24.8-25.3)	1.4 (1.0-1.8)	4.1 (3.2-5.3)	46.0 (42.0-50.2)	38.3 (4.5-42.3)	9.1 (7.2-11.1)	0.9 (0.6-1.4)	0.2 (0.1-0.3)
Latin America and the Caribbean	1980	5.5 (2.6-10.3)	23.5 (22.8-24.2)	5.8 (3.6-9.0)	9.2 (6.5-12.4)	52.2 (45.9-58.7)	26.9 (21.3-32.5)	5.4 (3.6-7.9)	0.4 (0.2-0.8)	0.0 (0.0-0.1)
Oceania	1980	6.4 (2.8-12.7)	22.6 (21.4-23.8)	3.2 (1.0-7.4)	7.5 (3.3-13.8)	57.9 (46.8-68.2)	25.3 (16.7-35.4)	5.4 (2.6-10.0)	0.5 (0.2-1.2)	0.1 (0.0-0.3)
South Asia	1980	3.8 (1.5-7.9)	19.2 (18.7-19.7)	33.0 (26.5-39.8)	22.0 (16.7-28.0)	39.2 (32.1-46.1)	5.5 (3.3-8.1)	0.3 (0.1-0.5)	0.0 (0.0-0.0)	0.0 (0.0-0.0)
Sub-Saharan Africa	1980	2.7 (1.0-5.8)	19.8 (19.0-20.5)	18.8 (13.4-24.5)	22.9 (17.5-28.7)	49.9 (43.4-56.4)	7.8 (5.0-11.8)	0.6 (0.3-1.1)	0.0 (0.0-0.1)	0.0 (0.0-0.0)
Central and eastern Europe	2012	7.5 (4.8-10.7)	26.5 (26.0-26.9)	0.8 (0.5-1.2)	2.4 (1.8-3.2)	35.8 (32.3-39.3)	41.3 (37.7-44.8)	15.7 (13.2-18.4)	3.3 (2.3-4.6)	0.7 (0.4-1.1)
Central Asia and north Africa-Middle East	2012	12.9 (10.3-15.9)	26.4 (26.2-26.6)	2.4 (1.9-3.0)	4.0 (3.2-4.8)	36.3 (33.4-39.0)	.7 (35.0-40.6)	15.2 (13.2-17.4)	3.4 (2.6-4.3)	1.0 (0.7-1.4)
East and south east Asia	2012	8.7 (6.1-12.2)	23.4 (23.2-23.6)	6.9 (5.6-8.4)	11.1 (9.2-13.3)	52.7 (49.1-56.1)	24.8 (21.6-28.1)	4.1 (3.1-5.3)	0.3 (0.2-0.5)	0.1 (0.0-0.1)
High-income Asia Pacific	2012	8.5 (6.2-11.2)	23.8 (23.6-24.0)	3.7 (2.8-4.8)	7.7 (6.2-9.5)	56.8 (53.3-60.1)	27.7 (24.7-31.1)	3.8 (2.9-4.9)	0.2 (0.1-0.4)	0.0 (0.0-0.0)
High-income western countries	2012	7.2 (5.5-9.2)	27.7 (27.5-27.9)	0.5 (0.3-0.6)	1.5 (1.2-1.8)	28.9 (26.5-31.2)	42.1 (39.6-44.7)	18.8 (16.7-21.0)	5.7 (4.5-7.0)	2.6 (1.8-3.5)
Latin America and the Caribbean	2012	8.6 (6.2-11.6)	26.4 (26.2-26.6)	1.6 (1.2-2.1)	3.5 (2.8-4.3)	36.9 (34.0-9.9)	39.1 (36.1-42.0)	14.8 (12.7-17.0)	3.3 (2.5-4.2)	0.8 (0.6-1.2)
Oceania	2012	15.1 (9.3-22.7)	25.6 (24.9-26.3)	1.4 (0.5-3.0)	3.8 (1.8-6.5)	44.7 (36.9-52.2)	32.1 (25.2-39.4)	13.8 (8.8-19.7)	3.0 (1.6-5.4)	1.3 (0.6-2.7)
South Asia	2012	9.3 (6.2-13.2)	21.7 (21.4-21.9)	22.9 (19.2-26.9)	15.7 (12.9-18.8)	44.3 (40.2-48.6)	14.6 (11.9-17.6)	2.2 (1.6-3.0)	0.2 (0.1-0.3)	0.1 (0.0-0.1)
Sub-Saharan Africa	2012	6.5 (4.7-8.5)	22.2 (22.0-22.5)	12.8 (10.6-15.2)	16.0 (13.7-18.6)	51.7 (48.5-54.8)	15.5 (13.3-17.8)	3.1 (2.4-4.0)	0.5 (0.4-0.7)	0.3 (0.2-0.4)

Countries

We grouped linked cancer and diabetes data on countries into 21 geographical sub-regions: high-income English-speaking countries; south Asia; central Asia; southeast Asia; east Asia; high-income Asia Pacific; central Africa; central Europe; southwestern Europe; northwestern Europe; eastern Europe; Middle East and north Africa; east Africa; west Africa; southern Africa; southern Latin America; Andean Latin America; central Latin America; Polynesia and Micronesia; Melanesia; and the Caribbean. These were then further grouped into nine geographical regions: sub-Saharan Africa (eastern, middle, southern, and western Africa); Latin America and the Caribbean (central and southern Latin America and the Caribbean); east and southeast Asia (eastern Asia); south Asia; high-income Asia Pacific; central Asia, Middle East and north Africa; central and eastern Europe; high-income western countries; and Oceania (including Polynesia and Micronesia). This is outlined in Appendix Table 4.

Appendix Table 4. Super-region, regions and countries with diabetes and BMI prevalence estimates.

Region	Sub-region
**Sub-Saharan Africa (48)**	**Central Africa (6)**: Angola, Central African Republic, Congo, DR Congo, Equatorial Guinea, Gabon
	**East Africa (17)**: Burundi, Comoros, Djibouti, Eritrea, Ethiopia, Kenya, Madagascar, Malawi, Mauritius, Mozambique, Rwanda, Seychelles, Somalia, Sudan, Tanzania, Uganda, Zambia
	**Southern Africa (6)**: Botswana, Lesotho, Namibia, South Africa, Swaziland, Zimbabwe
	**West Africa (19)**: Benin, Burkina Faso, Cabo Verde, Cameroon, Chad, Cote d'Ivoire, Gambia, Ghana, Guinea, Guinea Bissau, Liberia, Mali, Mauritania, Niger, Nigeria, Sao Tome and Principe, Senegal, Sierra Leone, Togo
**Central Asia, Middle East and North Africa (28)**	**Central Asia (9)**: Armenia, Azerbaijan, Georgia, Kazakhstan, Kyrgyzstan, Mongolia, Tajikistan, Turkmenistan, Uzbekistan
	**Middle East and North Africa (19)**: Algeria, Bahrain, Egypt, Iran, Iraq, Jordan, Kuwait, Lebanon, Libya, Morocco, Occupied Palestinian Territory, Oman, Qatar, Saudi Arabia, Syrian Arab Republic, Tunisia, Turkey, United Arab Emirates, Yemen
**South Asia (6)**	**South Asia (6)**: Afghanistan, Bangladesh, Bhutan, India, Nepal, Pakistan
**East and Southeast Asia (16)**	**East Asia (4)**: China, China (Hong Kong SAR), North Korea, Taiwan
	**Southeast Asia (12)**: Brunei Darussalam, Cambodia, Indonesia, Lao PDR, Malaysia, Maldives, Myanmar, Philippines, Sri Lanka, Thailand, Timor-Leste, Viet Nam
**Oceania (17)**	**Polynesia and Micronesia (13)**: American Samoa, Cook Islands, French Polynesia, Kiribati, Marshall Islands, Micronesia (Federated States of), Nauru, Niue, Palau, Samoa, Tokelau, Tonga, Tuvalu
	**Melanesia (4):** Fiji, Papua New Guinea, Solomon Islands, Vanuatu
**High-income Asia Pacific (3)**	**High-income Asia Pacific (3)**: Japan, Singapore, South Korea
**Latin America and Caribbean (35)**	**Andean Latin America (3)**: Bolivia, Ecuador, Peru
	**Caribbean (18)**: Antigua and Barbuda, Bahamas, Barbados, Belize, Bermuda, Cuba, Dominica, Dominican Republic, Grenada, Guyana, Haiti, Jamaica, Puerto Rico, Saint Kitts and Nevis, Saint Lucia, Saint Vincent and the Grenadines, Suriname, Trinidad and Tobago
	**Central Latin America (9)**: Colombia, Costa Rica, El Salvador, Guatemala, Honduras, Mexico, Nicaragua, Panama, Venezuela
	**Southern Latin America (5)**: Argentina, Brazil, Chile, Paraguay, Uruguay
**High-income Western countries (27)**	**High-income English-speaking countries* (6)**: Australia, Canada, Ireland, New Zealand, United Kingdom, United States of America
	**North Western Europe (12)**: Austria, Belgium, Denmark, Finland, Germany, Greenland, Iceland, Luxembourg, Netherlands, Norway, Sweden, Switzerland
	**South Western Europe (9)**: Andorra, Cyprus, France, Greece, Israel, Italy, Malta, Portugal, Spain
**Central and Eastern Europe (20)**	**Central Europe (13)**: Albania, Bosnia and Herzegovina, Bulgaria, Croatia, Czech Republic, Hungary, Macedonia (TFYR), Montenegro, Poland, Romania, Serbia, Slovakia, Slovenia
	**Eastern Europe (7)**: Belarus, Estonia, Latvia, Lithuania, Moldova, Russian Federation, Ukraine

References:

1. Food, Nutrition, Physical activity, and the Prevention of Breast Cancer. *Continuous Update Project Report: World Cancer Research Fund / American Institute for Cancer Research* 2010.
2. Food, Nutrition, Physical activity, and the Prevention of Colorectal Cancer. *Continuous Update Project Report: World Cancer Research Fund / American Institute for Cancer Research* 2011.
3. Food, Nutrition, Physical activity, and Gallbladder Cancer. *Continuous Update Project Report: World Cancer Research Fund / American Institute for Cancer Research* 2015.
4. Food, Nutrition, Physical activity, and the Prevention of Pancreatic Cancer. *Continuous Update Project Report: World Cancer Research Fund / American Institute for Cancer Research* 2012.
5. Food, Nutrition, Physical activity, and the Prevention of Endometrial Cancer. *Continuous Update Project Report: World Cancer Research Fund / American Institute for Cancer Research* 2013.
6. Food, Nutrition, Physical activity, and the Prevention of Ovarian Cancer. *Continuous Update Project Report: World Cancer Research Fund / American Institute for Cancer Research* 2014.
7. Food, Nutrition, Physical activity, and kidney Cancer. *Continuous Update Project Report: World Cancer Research Fund / American Institute for Cancer Research* 2015.
8. Food, Nutrition, Physical activity, and Liver Cancer. *Continuous Update Project Report: World Cancer Research Fund / American Institute for Cancer Research* 2015.
9. Food, Nutrition, Physical activity, and Stomach Cancer. *Continuous Update Project Report: World Cancer Research Fund / American Institute for Cancer Research* 2016.
10. Food, Nutrition, Physical activity, and Oesophageal Cancer. *Continuous Update Project Report: World Cancer Research Fund / American Institute for Cancer Research* 2016.
11. Tsilidis KK, Kasimis JC, Lopez DS, Ntzani EE, Ioannidis JPA. Type 2 diabetes and cancer: umbrella review of meta-analyses of observational studies. *BMJ* 2015; **350**.
12. Renehan AG, Tyson M, Egger M, Heller RF, Zwahlen M. Body-mass index and incidence of cancer: a systematic review and meta-analysis of prospective observational studies. *The Lancet* 2008; **371**(9612): 569-78.
13. Kyrgiou M, Kalliala I, Markozannes G, et al. Adiposity and cancer at major anatomical sites: umbrella review of the literature. *Bmj* 2017; **356**: j477.
14. Forman D, Brewster D, Gombe Mbalawa C, et al. Cancer Incidence in Five Continents, Vol. X (electronic version). *IARC; Lyon* 2013.
15. Ezzati M, Lopez AD, Rodgers A, Vander Hoorn S, Murray CJL. Selected major risk factors and global and regional burden of disease. *The Lancet* 2002; **360**(9343): 1347-60.
16. NCD Risk Factor Collaboration (NCD-RisC). Worldwide trends in diabetes since 1980: a pooled analysis of 751 population-based studies with 4.4 million participants. *The Lancet*; **387**(10027): 1513-30.
17. NCD Risk Factor Collaboration (NCD-RisC). Worldwide trends in children's and adolescents' body mass index, underweight, overweight and obesity, in comparison with adults, from 1975 to 2016: a pooled analysis of 2,416 population-based measurement studies with 128.9 million participants. *Lancet* In Press.

18. NCD Risk Factor Collaboration (NCD-RisC).. Trends in adult body-mass index in 200 countries from 1975 to 2014: a pooled analysis of 1698 population-based measurement studies with 19·2 million participants. *The Lancet*; **387**(10026): 1377-96.
